# Life Cycle and Immature Stages of the Arctiid Moth, *Phoenicoprocta capistrata*

**DOI:** 10.1673/031.008.0501

**Published:** 2008-01-25

**Authors:** Laura Rodríguez-Loeches, Alejandro Barro

**Affiliations:** Departamento de Biología Animal y Humana, Facultad de Biología de la Universidad de La Habana. Calle 25, # 455, entre J e I, CP 10400, Plaza de la Revolución, Ciudad de La Habana, Cuba

**Keywords:** Dyar's rule, female limited polymorphism, color morphs, Lepidoptera, Arctiidae

## Abstract

*Phoenicoprocta capistrata* (Fabricius 1775) (Lepidoptera: Arctiidae) is an arctiid moth reported for the Caribbean and Brazil, whose immature stages and life cycle are unknown. In this study, and for the first time, a host plant is registered and the immature stages and the captivity life cycle are described using a Cuban population. Larvae feed on fowlsfoot, *Serjania diversifolia* (Jacq.) Radlk (Sapindales: Sapindaceae). One complete cohort was obtained from December of 2004 to February of 2005 and about 57 days lapsed from oviposition to adult emergence. The egg is light green-yellowish and semi-spherical. Most larvae developed through 6 or 7 instars, although there were individuals with 8 instars. The last instar has a cephalic capsule width of 2.04 ± 0.06 mm (n = 29) irrespective of the number of instars. The cephalic capsule growth curves of the larvae with 6 and 7 instars have different slopes, but both follow a geometric pattern consistent with the Dyar's rule. In each larval molt the setae types and the larvae coloration change. Adult females have two color morphs, one orange-reddish and the other blue. Female descendants of blue and red females differ in the proportion of color morphs, which could indicate the existence of a female-limited polymorphism phenomenon in this species.

## Resumen

*Phoenicoprocta capistrata* (Fabricius 1775) (Lepidoptera: Arctiidae) es un árctido registrado para el Caribe y Brasil, cuyos estadios inmaduros y ciclo de vida son desconocidos. En este estudio se registra por primera vez una planta hospedera para esta especie y se describen sus estadios inmaduros y ciclo de vida en cautiverio usando una población de Cuba. Las larvas se alimentan de la liana, *Serjania diversifolia* (Jacq.) Radlk (Sapindaceae). Se obtuvo una cohorte completa desde diciembre de 2004 hasta febrero de 2005, transcurriendo alrededor de 57 días desde la oviposición hasta la emersión de los adultes. El huevo es verde amarillento y semiesférico. La mayoría de las larvas se desarrollan en 6 ó 7 instares, aunque algunos individuos se desarrollaron en 8 instares. El último instar larval tiene un ancho cefálico de 2.04 ± 0.06 mm (n = 29) independientemente del número de instares. Las curvas de crecimiento de la cápsula cefálica de las larvas de 6 y 7 instares presentan pendientes diferentes, pero ambas siguen un patrón geométrico consistente con la regla de Dyar. En cada muda larval se producen cambios en el tipo de setas y en la coloración de las larvas. Las hembras adultas presentan dos morfos de coloración, uno naranja rojizo y el otro azul. Las hembras descendientes de hembras azules y rojas difieren en cuanto a la proporción de morfos de coloración, lo que podría indicar la existencia del fenómeno de polimorfismo limitado al sexo femenino en esta especie.

## Introduction

Arctiid moth species of the Neotropical genus *Phoenicoprocta* Druce are distributed within the Caribbean region in the islands of St. Tomas, Bahamas, Puerto Rico, Jamaica, Virgin Islands, Haiti, St. Croix and Cuba. Of the 4 species of this genus reported for the Caribbean, *Phoenicoprocta capistrata* (Fabricius) has the widest distribution and occurs in Brazil, Cuba, St. Tomas, St. Croix and Puerto Rico. Forbes (1930) stated that some species of this genus are extraordinarily variable. *Phoenicoprocta capistrata* is not an exception; adults of this species have a highly coloured body and there are two female colour morphs, one orange-reddish and the other metallic blue. The latter is less common and formerly named *P. capistrata* var. *eximia* (Herrich-Shäffer). We have not found information in the literature about the immature stages, life cycle and host plants of P. *capistrata*. In the present study we describe the immature stages of *P. capistrata* and its life cycle in captivity using a Cuban population. We also report a host plant for this species.

## Materials and Methods

### Collecting and Rearing

*P. capistrata* larvae were first collected on *S. diversifolia* in mid-November of 2004 at the town “La Salud”, La Habana, Cuba, located at 22° 46′ oo″ latitude and -82° 32′ oo″ longitude. Larvae were reared in captivity at the Facultad de Biología de la Universidad de La Habana, under natural photoperiod, relative humidity and temperature. When adults emerged their identification was checked using the original description of Fabricius (1775) and adult voucher specimens were placed in the Insect Collection at the Facultad de Biología de la Universidad de La Habana. Adults were placed together for mating in cylindrical plastic containers (27 cm height × 30 cm diameter) with gauze as lid and were provided with fresh saturated sucrose solution. Females were isolated after the first copulation and eggs laid were used for further analysis.

Newly hatched larvae were placed in plastic dishes (35 × 10 mm) until they reached sufficient size to be reared in glass Petrie dishes (100 × 10 mm). They were provided with fresh host plant leaves daily until the larval stage finished. Larvae that hatched at the same day were reared in groups of up to 30 larvae. When a larva showed desynchronised development in relation to the rest, it was isolated in a different container in order to study the individual variations in stage duration. The progeny of individual females were marked and reared in separate dishes to analyze the female morph frequency in the descendants. We obtained one complete laboratory cohort from December 2004 to February 2005. Minimum daily temperature fluctuated between 17.4°C and 27.4°C and the relative humidity varied from 80 to 100%. Both abiotic variables were measured using a psychrometer with a precision of up to 0.2 °C.

### Life cycle

Eggs obtained from the laboratory colony were observed daily to determine the duration of this stage. The proportion of infertile eggs was also recorded.

To establish the duration of the different larval instars, containers with larvae were daily checked for larval head capsule exuviae, which indicate moulting. The width of the head capsule exuvia was measured using an ocular micrometer attached to an Olympus 220465 stereomicroscope with a precision of 50 µm per division. We use the width of the head capsule exuvia as an equivalent of the head width in caterpillars as we noticed that these larvae do not deform the head capsule when moulting, except in the larval-pupal moult. For that reason we measured the head capsule width of the last instar directly on the larvae. This variable was used as a linear measurement of size in larvae and it was used to test Dyar's rule in this moth species. Dyar's rule (Dyar 1890) states that larval growth progresses geometrically and by a relatively constant factor. Dyar's rule predicts that a linear measure of size increases by a constant factor from one instar to the next. According to Dyar's rule, a strong linear relationship is predicted between the log of a linear measurement of size and instar number.

Larvae that pupated the same day were placed together for studying the duration of the pupal stage. At emergence, the sex and colour morph of the adults was recorded for detecting possible differences in the pupal stage duration related with sex.

For females that copulated only once in their life, we examined the following characteristics of the oviposition: number of days lapsed between mating and the first oviposition, number of days dedicated to oviposition events and total number of eggs laid per individual in the whole oviposition period.

### Describing immature stages

For analysing the dimension and form of the eggs, two base diameters and height were measured using the same ocular micrometer attached to the OLYMPUS stereomicroscope mentioned before, but using a precision of 25 µm per division. Surface relief and micropilar details were photographed and processed using the system of digital cameras of a Digital Motic DMB microscope (Motic, Tachlovice, CZ) with a magnification of 40 × coupled to a computer running the digital image software Motic Image 2000 version 2.1 for Windows.

As larvae may have 6, 7 or 8 instars we decided to describe larval stages of those larvae with 7 instars, which were 60% of the 49 larvae whose life cycle was studied.

Morphological terminology follows that used by Stehr (1987) and Scoble (1992). Photographs of larvae, pupae and adults were taken with the macro mode of a Casio QV-4000 (4.1 megapixels) digital camera (Casio, Tokyo, Japan), although detailed photographs were taken using the magnification of up to 40X of a stereomicroscope coupling the lens of the camera directly to the ocular of the microscope.

The length of individuals after hatching, as well as the maximum length of 2nd instar larvae was measured using the same ocular micrometer attached to the Olympus stereomicroscope (http://microscope.olympus.com/) with a precision of 50 µm per division. The maximum length of the larvae from third to seventh instar was measured using a rule with a precision of 1 mm. Pupal weight was measured using an analytical scale Mettler H2o (Mettler-Toledo http://us.mt.com) with a precision of 0.01 mg. Pupal length and width were measured using a rule with a precision of 1.0 mm.

For all larval instars, one specimen was killed in boiling water and preserved in 70% alcohol with its corresponding head capsule in the Entomological Collection at the Facultad de Biología de la Universidad de La Habana. One pupal specimen, cocoons and adults of both sexes and colour morph were also deposited in this collection.

### Statistical analysis

Statistical analyses were performed using STATISTICA 5.1 for Windows. Variables were subjected to Shapiro-Wilks' *W* test for normality and Levene test for variance homogeneity. Almost all comparisons were performed using the Mann-Whitney two-tailed non-parametric test, since almost all the variables do not follow a normal distribution. The parametric t-test was used to compare two base diameters of the egg. The regression line equations of the log cephalic capsule width versus instar number were determined using a simple linear regression analysis. The simple linear regression analysis and the analysis of covariance were also performed using GraphPad Prism 4.03 (www.graphpad.com). The significance level (p) for all tests was fixed at p<0.05.

## Results

### Host Plant

In the field, the caterpillars were found feeding on the Neotropical fowlsfoot, *S. diverifolia* (Jacq.) Radlk (Sapindales: Sapindaceae). First instar larvae feed on the epidermis of young fresh leaves while the rest of the larvae feed on mature leaves but not on its veins.

### Life Cycle

**Figure 1.**
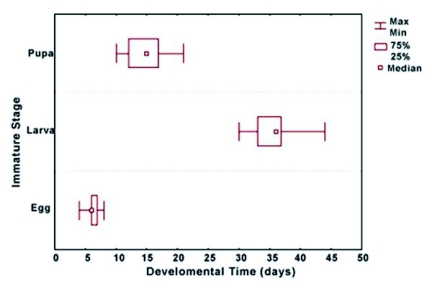
Characteristic duration of the different immature stages of *Phoenicoprocta capistrata* expressed as the median value of 193 eggs, 49 larvae and 90 pupae. The 25 and 75% percentils and the minimum and maximum values are shown.

Under laboratory conditions, eggs finished their development on the 6^th^ day after opposition, larvae begin to prepupate about 36 days after hatching and pupae ended their development about 15 days later (Figure 1).

**Figure 2.**
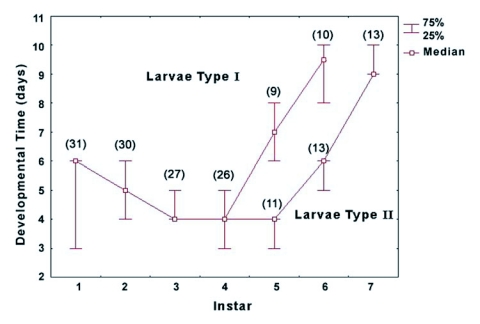
Developmental time of each instar for *Phoenicoprocta capistrata* larvae of Type I (larvae that finish development in 6 instars) and Type II (larvae that finish development in 7 instars). At the 5^th^ instar the developmental pathways diverge showing a more abrupt daily increase for larvae that will have a 6 instar. The first 4 points of the curve represent the median values including the two types of larvae since no statistical difference (1<p<0.07) was found for the first 4 larval instars. The numbers in brackets represents the number of individuals analyzed.

Most of the 49 larvae developed through 6 instars (defined as Type I larvae) (n = 17) or 7 instars (defined as Type II larvae) (n = 29). However, there was no difference in total developmental time between them (p = 0.17), due to statistically significant differences in the duration of the 5 (p = 0.0005) and the 6^th^ instars (p = 0.0002) of both types of larvae (Figure 2). There were no significant statistical differences in duration of the 1^st^, 2^nd^, 3^rd^ or 4^th^ instars of both types of larvae (p >0.05). Three larvae of 49 developed through 8 instars, which was a rare condition in this cohort, and they were not included in the analysis.

**Figure 3.**
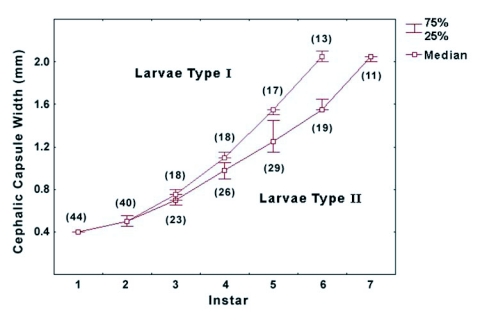
Cephalic capsule width of each instar for *Phoenicoprocta capistrata* larvae of Type I (larvae that finish development in 6 instars) and Type II (larvae that finish development in 7 instars). At the 3^rd^ instar the developmental pathways diverge showing a more abrupt increase in width for larvae that will have a 6^th^ instar (Type I), but the last instar of both type of larva have a similar head width. The first 2 points of the curve represent the median values including the two types of larvae since no statistical difference was found for the first 2 larval instars. The numbers in brackets represents the number of individuals analyzed.

The cephalic capsule width of larvae Type I and II (Figure 3) did not differ in the 1^st^, 2^nd^ and their respective last instar (o >0.18), but did differ in the rest of the instars. The difference in the cephalic capsule width of the third instar was significant (p = 0.006) and represents the first sign of change in the developmental pathway of both types of larvae. This difference is strong and more robust in the fourth, fifth and sixth instars (p <0.0001).

Considering the change of cephalic capsule width, larval growth of *P. capistrata* seems to follow a geometric pattern consistent with Dyar's rule (Dyar 1890), on the base of the growth of cephalic capsule width. The regression lines for both types of larvae have a high determination coefficient and the curves have statistically different slopes (F = 20.5, p = 0.001). The regression line equation for Type I larvae was (y = - 0.5656 + 0.1483x; R^2^ = 0.9949) and for Type II larvae it was (y = - 0.5185 + 0.1201x; R^2^ = 0.9957).

Larvae stay in the prepupal wandering stage for about 2 days and then enter the pupal stage. The pupae weighed 58.5 ± 9.9 mg (n = 20) and had a maximal width of 3.0 ± 0.1 mm (n = 64) and a length of 8.6 ± 0.5 mm (n = 64). There is a statistically significant difference in the duration of pupal stage between sexes (p <0.0001); females had a shorter duration (15 ± 2 days, n = 41) than males (17 ± 2 days, n = 32). Pupal mortality was about 11% of the 83 pupae studied.

Adult eclosion occured any time between 6 and 1 hours before sunset. Mating occurs at dusk. All females (n = 11) engage in calling position the same day of their emergence and 15 minutes before sunset. Females more than one day old always showed the calling position anytime between 1 and no minutes before sunset (n = 26). Copulation occured in the time span between 45 minutes before to 20 minutes after sunset (n = 25), and 52% of them occurred in the period of ± 5 minutes of sunset. Pairs remain in copula for 305 ± 70 min (n = 24). Females began to oviposit the day after copulation occurred and could oviposit during each of the next 3 days, but most of the eggs were laid during the first two days after mating. The total number of eggs laid by a female after copulation averaged 51 ± 21 eggs (n = 8). The number of non-fertilised eggs per female varies from 0 to 9% (n = 6 females).

**Figure 4.**
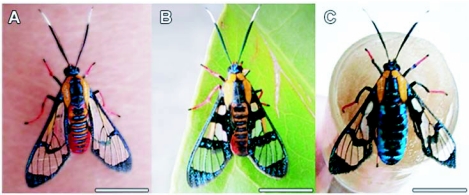
*Phoenicoprocta capistrata* adults. A: Male. B: Female reddish morph. C: Female blue morph. The scale bars represents 7 mm.

**Figure 5.**
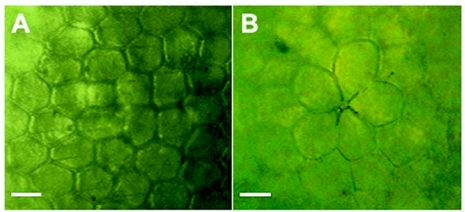
Microphotograph of the details of the chorion of the egg of *Phoenicoprocta capistrata*. The scale bar represents 20 µm

*P. capistrata* is a species with two female color morphs, the orange-reddish morph was similar to the male and the blue morph was completely different from the male (Figure 4). The female descendants of 4 red females were all red, but the female descendants of 2 blue females were 50% blue and 50% red.

### Description of Immature Stages

In the laboratory females laid eggs singly or in small clusters of up to 30 eggs, generally on the undersurface of the smooth lid covering the top of the cage. The eggs were tightly attached to the substrate and when they were laid in batches they were not in contact with one another. The eggs were upright and were hemispherical in form; no statistical difference was found between the two perpendicular base diameters (t = 1.55, p = 0.13, n= 20), and its height was of 0.67 ± 0.05 mm (n = 20), which represents 3/4 of the base diameter (0.86 ± 0.04 mm, n = 20). The eggshell was shiny and light green-yellowish in colour, turning more yellow at the end of its development. The chorion was hexagonally and pentagonally reticulated except for the micropylar area, which was rosette-like and was composed of 5 (n = 6) or 4 (n = 3) petaloid cells (Figure 5).

**Figure 6.**
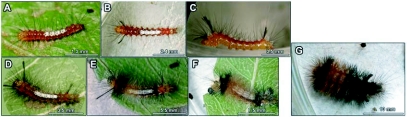
Photograph of the diferents larval stages of typical *Phoenicoprocta capistrata* individuals with 7 instars. All individuals are shown in dorsal view excpet for C with a lateral view. A: 1^st^ instar, B: 2^nd^ instar, C: 3^rd^ instar, D: 4^th^ instar, E: 5^th^ instar, F: 6^th^ instar, G: 7^th^ instar.

Newly hatched first instar larvae (Figure 6A) had a mean length of 2.68 ± 0.19 mm (n = 29) and a cephalic capsule width of 0.40 ± 0.01 mm (n = 29). The general body colour was greenish-yellow, with a greenish-white middorsal longitudinal stripe from A3 to A6 abdominal segments. The head was yellow but the stemma and mandibles were black. The body of larvae was covered by chalazae bearing one long filiform and dark brown seta except for T2 and T3 that have two dorsal pinacula each bearing 3 setae, and T1 that is covered by 6 anterior dorsal pinacula each with a single seta. When larvae feed, the thorax and the abdomen turn red except for the thoracic segment Ti that is yellow with the anterior margin scattered with red speckles and the ventral surface of the last abdominal segments that is reddish-yellow. In these fed larvae, the middorsal A3–A6 longitudinal stripe was white and the base of the dorsal chalazae in these segments was reddish-brown. In the other segments, the dorsal anterior chalazae were white while the posteriors were brown. Legs and prolegs were reddish-yellow.

Second instar larvae (Figure 6B) eat the moulted skin as many other instars do. They had an average length of 4.84 ± 0.36 mm (n = 27) in length and a cephalic capsule width of 0.52 ± 0.03 mm (n = 29). The head was similar to that in the first instar, but with brown ecdysial sutures. The main change in this instar was the appearance of verrucae covering the body instead of chalazae, and the appearance of one black tuft at each side of the anterior margin of T3 and A8, pointing diagonally forwards at T3 and diagonally backwards on A8. The colour of the thorax varied individually from reddish-yellow with red rings around verrucae to a completely red coloration. Abdominal segments were red, except for A9 and A10, which were yellow with red rings around verrucae, and the middorsal white stripe A3-A6. Segments A7 and A8 had a thin middorsal longitudinal yellow line. The lateral surface was marked with a discontinuous subspiracular longitudinal white stripe extending from the anterior margin of A3 to the anterior margin of A9. The ventral surface was yellow scattered with a few scattered red speckles. The prolegs had a square brown spot on its outer surface.

Third instar larvae (Figure 6C) had an average length of 7 ± mm (n = 28) and a cephalic capsule width of 0.70 ± 0.08 mm (n = 23). The colour pattern was basically the same as the second instar, but tufts were longer and one dark red subdorsal stripe was present on each side of the middorsal white stripe. These new stripes had the same extension of the middorsal stripe but were thinner. There were some individuals with yellow ventral and subventral regions and others with these regions in pale red. The discontinuous subspiracular longitudinal white stripe extended from the posterior margin of A1 to the anterior margin of A9.

Fourth instar larvae (Figure 6D) had an average length of 7 ± mm (n = 26) and a cephalic capsule width of 0.98 ± 0.08 mm (n = 26). It was very similar to the third instar. Tufts were longer and the two subdorsal stripes were darker than those in the 3^rd^ instar larvae. The head had great colour variability; in some individuals it was completely orange while in others it was pale yellow with different degrees of black pigmentation that could be only around stemma or may extend to the whole head up to the epicranial suture. The first antennal segment was white and the rest were yellow. The labrum was white spotted. Thoracic segments and abdominal A9 and A10 were yellow, but the rest of the segments were red-brownish. The middorsal white stripe extended to the anterior part of A7 but it was narrower in this segment. Most of the setae were black and they were more abundant in relation to those present in the previous instar.

Fifth instar larvae (Figure 6E) had an average length of 11 ± 1 mm (N=29) and a cephalic capsule width of 1.39 ± 0.16 mm (n = 29). The head was black with brown sutures and dark brown stemma but the clypeus and the labrum were white spotted. The thorax was dark yellow except for T3 and the intersegmental space T3-A1 which were black. Most of the abdominal segments were reddish-brown, except for A1 and A7 which were black, and A8, A9 and A10 which were reddish-yellow. Two longitudinal and discontinuous brown lines were present, one at both sides of the middorsal line and included within the middorsal white stripe, giving the appearance of two dashed brown lines within the white middorsal stripe. The dark red subdorsal stripes turned darker and wider and extended up to A7. The anterior verrucae from A3 to A6 had numerous white setae. The setae of T2, T3, A8 and A9 were longer than the setae of the other segments.

Sixth instar larvae (Figure 6F) had an average length of 15 ± 2 mm (N=22) and a cephalic capsule width of 1.59 ± 0.08 mm (n = 19). The number of setae was notably increased and tufts disappeared. Those that in the previous instars were white were orange-yellowish. Segments A8 and A9 may have different degrees of black pigmentation varying from only a few black spots to completely black. The rest of the segments maintained the same general colour pattern of the previous instar.

The seventh instar (Figure 6G) had an average length of 20 ± 2 mm (n = 29) and a cephalic capsule width of 2.04 ± 0.06 mm (n = 29). The head was black with brown sutures except for the adfrontal suture that was black. Mouth pieces, labrum and antenna were similar to those in fifth instar larvae. The thoracic segment Ti was completely pale yellow but T2 and T3 had one transverse black band. The first abdominal segment was black with one dense and short black tuft verricule-form at each lateral side pointing backward; these structures were also present in A7. The middorsal longitudinal white stripe that was present in all previous instars was lacking. From A2 to A4 the thoracic colour pattern inverted; intersegmental spaces were black and the transverse band was dark yellow but individual variation was high, in some cases the yellow color only surrounds and colors the verrucae and there are black spaces between verrucae, while in other individuals the black extended over the whole area. The abdominal segments A5 and A6 were black with dark yellow around verrucae, but some individuals could have this segment entirely black. From segments A7 to A10 the dorsal coloration was black. Lateral coloration was marked by the discontinuous subespiracular longitudinal stripe, which was pale yellow in this instar. Below and above this stripe the coloration was pale orange except for A1 and A7 which were black above the stripe. The ventral surface was ashy yellow. Legs and prolegs were dark yellow and crochets were brown and arranged in a heteroideous mesoseries. There were two black ventral verrucae on Ai. Dorsal verrucae were dense and reddish-brown in those segments with dark yellow coloration and black in those with black coloration. There were also a few white setae scattered throughout the body. On the central verrucae of T2 and T3 there was one white setae longer than the rest and pointing forward. Spiracles were pale brown with the border and central areas black. Some individuals had a more orange body. T3 and Ai were orange instead of black and with more orange setae than black.

**Figure 7.**
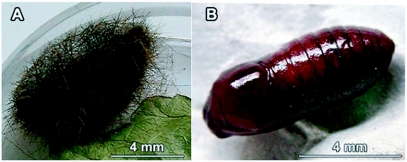
Photograph of the coocon (A) and pupa (B) of *Phoenicoprocta capistrata*.

In the prepupal stage the mature larvae stoped feeding and began to search for a site to pupate. It then shrank and detached the setae and began the construction of a cocoon with silk and its setae. This process lasted about two days. The denuded larva had a light yellow colour, which was conserved during metamorphosis and at the beginning of the pupal stage.

The pupa was obtect and enclosed in a thin flimsy and dark cocoon composed of the broken off setae of the caterpillar (Figure 7A). Pupae weight was 58.5 ± 9.9 mg (n = 20) with a length of 8 ± 1 mm (n = 64) and a width of 3 ± mm (n = 64). It had a shiny reddish-brown surface (Figure 7A) with only a few black spots but later in development it had dark irregular spots all over the body. The surface was covered with a few setae along the lateral line near the spiracles, over the dorsum of the head near the antennas and there was also a line of setae on both sides of the anterior region of the prothorax and a few scattered setae over the mesothorax. There were moderately dense punctures on abdominal segments, except for the zone nearby the anal and genital regions as well as in the zone surrounding the posterior margin of the abdominal segments. The spiracles and the division lines of the different parts of the body were black. At ventral view, the posterior wing edge reach the A4 segment. Labial palps were short and triangular. The femur of prothoracic legs were not visible. Mesothoracic legs did not meet midventrally. Flanged plates were absent. The cremaster was weak and it was composed of a few translucent hook-like setae only visible with a magnification about 40X.

## Discussion

### Host Plant and Immature Stages

To our knowledge *S. diversifolia* is the first report of a host plant for *P. capistrata*. The databases HOSTS (Robinson et al. 2000, discussed by Robinson 1999) and Janzen and Hallwachs (2005) do not register any host plant record for *P. capistrata*. However, HOSTS does record *Manihot esculenta* Crantz (Euphorbiaceae) as the host plant for *Phoenicoprocta sanguinea* (Walker), which is the same hostplant reported by Forbes (1930) for *Phoenicoprocta vacillons* (Drury). *Phoenicoprocta* species recorded in Janzen and Hallwachs database are the polyphagous caterpillars *Phoenicoprocta paucipuncta* Dyar and *Phoenicoprocta biseriatum* -still *Cosmosoma biseriatum* Schaus-, both were reported to feed on Fabaceae, Malpighiaceae and Meliaceae species. *Cosmosoma biseriatum* is being revised because it possibly belongs to the genus *Phoenicoprocta* (Daniel Janzen, Department of Biology, University of Pennsylvania, personal communication). It is interesting that this species also feeds on *Serjania atrolineata* Wright (Sapindaceae) a species belonging to the same genus of the host plant reported in this article for *P. capistrata*. Other arctiid species of the related genus *Cosmosoma* have been also reported to feed on *Serjania* species (Janzen and Hallwachs 2005). *Serjania* hostplants are interesting linking factors that could be helpful to clarify the taxonomic status of the species inside the genera *Phoenicoprocta* and *Cosmosoma* and it could be useful for discussing phylogenetic approaches on the complex relationships inside the Arctiidae family.

*Phoenicoprocta* species need complete revision and some species could change their current taxonomic status. The adults of *P. capistrata* (Fabricius 1775) have always been identified as such in Cuba and it is the only *Phoenicoprocta* species recorded so far for this territory. Its characteristics fit into the Fabricius (1775) description for *P. capistrata*, which we have consulted and by which we have identified the adults specimens used in our experiments. In order to avoid future confusion due to taxonomic changes, voucher specimens of the *P. capistrata* adults and its immature stages used in this research have been placed in the insect collection at the Facultad de Biología de la Universidad de La Habana.

In general, the morphology of the immature stages of *P. capistrata* is similar to that of other arctiids and it fits into the general arctiid pattern described by Stehr (1987) and Scoble (1992). One of the most interesting aspects in the anatomy of *P. capistrata* larvae is the occurrence of different types of setae arrangements, which appear in different larval instars. The first instar is only covered by chalazae bearing one long filiform seta and pinaculums bearing 1 or 3 setae. The intermediate instars are mainly covered by verrucae and tufts. A diagnostic character of the last instar is the presence of two dense and black verricles in the first and seventh abdominal segments.

### Life Cycle

Considering that we present here typical variation in temporal traits of the life cycle not under controlled abiotic conditions, comparing these results with others should be done with caution. Life cycle traits of lepidopterans are plastic. Time and size traits are affected by temperature, photoperiod, humidity, diet, density and host quality (Nylin and Gotthard 1998). The life cycle study described here was for insects in a laboratory culture, but abiotic conditions and density of individuals was not controlled as abiotic conditions were allowed to naturally fluctuate, as is typical of the transition through the months of December to February in Havana City. These months are the coldest and driest in Cuba, but with daily drastic changes. During the months of this study a variation in temperature about 10 °C (17.4 °C minimum – 27.4°C maximum) and about 20% in relative humidity (80–100%) were recorded. This temperature difference causes a variation of 7 days in the duration of embryonic development in another lepidopteran egg in a study that was carried out under controlled temperatures (Hansen et al. 2004). The temporal variations in our study could be the result of the uncontrolled abiotic and biotic conditions, which probably simulate the natural variations of a hypothetical natural *P. capistrata* population during these months. The fact that male pupal development was found to be longer than development of the female has also been reported for other arctiids (Otazo et al. 1984; Betzholtz 2003).

In this study the number of larval instars (6, 7 or more) varied. There can be up to 9 instars in individuals of one cohort subjected to the same variable conditions of this study (unpublished data). The number of larval instars for arctiids has been stated to be as low as 5 and as high as 7 for different species (Bratley 1932; Torre 1967; Otazo et al. 1984; Betzholtz 2003; Gomi et al. 2003), but always with only two alternatives. Most larvae of *P. capistrata* developed through 6 or 7 instars, which are inside the range stated for arctiids, but some larvae developed through 8 or 9 instars. The number of moults undergone by insects is affected by diet quality and quantity, temperature, humidity, photoperiod and also by sex (Sweetman and Whittemore 1937; Wigglesworth 1972; Daly 1985; Hutchinson et al. 1997). We did not design a specific experiment to evaluate the different factors affecting larval moults in *P. capistrata*. However we can not say that sex is the cause of the different alternatives because females could have 6 or 7 instars. Also, all larvae were subjected to the same variable temperature, humidity and natural photoperiod. Larvae were fed with the same host plant species so that the diet quality was the same for all larvae. But the diet quantity is one of the factors that could cause the plasticity in the number of instars. Although the daily amount of food was maximized, larval density varied from 1 to 8 larvae per Petri dish. As a result larval competition for food varied considerably. A genetic factor is also possible. The questions about which factors affect the number of larval instars in *P. capistrata*, and how the number varies between the individuals, remain opens.

One of the most intriguing results in this study is the fact that no matter the number of instars a larva has, it finishes its development with a cephalic capsule width of about 2 mm. We could verify the same pattern in larvae with 8 and 9 instars (unpublished data). The cephalic capsule width shows a geometric progression pattern of growth consistent with Dyar's rule (Dyar 1890), with an upper limit of about 2 mm but with different growth alternatives in instar number. Then a question arises: what determines the constant limit of the greatest cephalic capsule width in the larvae of this species? It is known that in holometabolus insects there is a threshold size which determines that a particular instar is the last (Nijhout 1975), and within this last instar, a critical weight determines when the metamorphosis larva-pupa occurs (D'Amico et al. 2001). Nijhout (1981) stated that there is a strong positive non-linear relationship between the head capsule width of the last larval instar and the critical weight. It seems that in *P. capistrata* larvae, the critical weight can only be reached with a cephalic capsule width of about 2 mm, which could constitute the threshold size in this species.

*P. capistrata* adults have a copulation duration of about 300 minutes, which is long in comparison with the copula of another arctiid moth, *Empyreuma pugione* (L.) that only lasts 25 minutes (Otazo et al. 1984). But it is not as long as the copula of the arctiid moth *Utetheisa ornatrix* (L.) that could last 550 to 720 minutes (LaMunyon and Eisner 1993, 1994). Copulation duration is a multifactor complex trait that varies between species and individuals. Factors affecting copulation duration can be categorized as independent and dependent of the reproductive strategy of males and females. Temperature (Scott 1972) and body size (LaMunyon and Eisner 1993; Charnov and Parker 1995; Parker et al. 1999) are two such factors. These factors affect the minimum time required for the successful transfer of sperm during copula but there is evidence that the spermatophore is passed early in the copula period in lepidopterans (Shields and Emmel 1973). Differences between species in the total copulation duration is induced more by those factors dependent on the reproductive strategy and fertilization success of both sexes for each arctiid species. For example, Charnov and Parker (1995) suggest that copulation duration increases with the time to find a female to mate with. Another criteria is that across species, the evolutionary stable ejaculate expenditure (therefore the copulation duration) should increase both with the risk of sperm competition (the average probability that the females in the population will mate with more than one male or the polyandry level) and the intensity of the sperm competition (the average number of males competing for a given set of ova) (*reviewed* inParker 1998). It is difficult to evaluate how these factors affect copulation duration in arctiid species without a clear panorama of their reproductive strategy. Four main hypotheses have been designed to explain the adaptive significance of prolonged copulations: the sperm removal hypothesis, cryptic female choice hypothesis, sperm loading hypothesis and in-copula guarding hypothesis *(all summarized* inGarcía-Gonzalez and Gomendio 2003). The in-copula guarding hypothesis suggests that remaining in copula may function as an extreme form of mate guarding if it prevents the female from remating before oviposition (Alcock 1994), thus possibly reducing or avoiding sperm competition from future ejaculates. This could be the reason why P. *capistrata* has longer copulation duration than expected by its size if it is compared with the shorter copulation duration and bigger size of *Empyreuma pugione*. Analysis of the reproductive strategies of males and females of these species is necessary to understand these relationships.

Although the number of females whose progeny was analyzed in relation to female morph proportion is still low, some evidence was presented for dependence of the colour of female progenitor and the morph proportion of its female descendants. This could indicate that this polymorphism in the females has a high genetic component that suggests that it is a case of female-limited genetic polymorphism. This type of polymorphism has received some attention in butterflies (Clarke et al. 1985; Cook et al. 1994; Nielsen and Watt 2000) but not in moths. Further experiments are needed to determine the genetic and evolutionary causes of this polymorphism in *P. capistrata*.
